# Idiopathic myositis ossificans of the deltoid muscle: A case report with unique presentation and MRI findings

**DOI:** 10.1016/j.ijscr.2020.02.043

**Published:** 2020-02-25

**Authors:** Mohammad M. Al-Qattan, Ahmad A. Al-Boukai, Yasir AlShehri, Yazeed A. AlSaadi, Hisham M.A. Elaaqip, Ali H. Alassiri

**Affiliations:** a Division of Plastic Surgery and Hand Surgery, Department of Surgery, King Saud University, Riyadh, Saudi Arabia; b Division of Plastic Surgery, Department of Surgery, National Guard Health Affairs, Riyadh, Saudi Arabia; c Department of Medical Imaging and Radiology, King Saud University, Riyadh, Saudi Arabia; d Division of Plastic Surgery, Department of Surgery, King Faisal Specialist Hospital and Research Center, Riyadh, Saudi Arabia; e Department of Surgery, Taif University, Taif, Saudi Arabia; f Department of Pathology and Laboratory Medicine, King Abdulaziz Medical City, King Saud Bin Abdulaziz University for Health Sciences, Riyadh, Saudi Arabia

**Keywords:** Myositis ossificans, Deltoid muscle, MRI

## Abstract

**Introduction:**

Myositis ossificans of the deltoid muscle is extremely rare. We present a case with unique presentation and MRI findings.

**Case Report:**

A 21-year-old female presented with a 3-month history of a swelling in lateral aspect of the right arm. There was no history of trauma, injection, or surgery to the area. Examination showed a 3 × 3 cm firm mass in the deltoid muscle. MRI findings were suggestive of myxoma. Surgical excision was done and histopathology showed the classic features of myositis ossificans. There was no recurrence at the final follow-up 7 months later.

**Discussion:**

Myositis ossificans of the deltoid muscle is very rare with only 6 cases previously reported in the literature. Our case is unique in presentation because it is the first case reported with no history of trauma, Injection, sickness or surgery in the area. We also review the literature for MRI features of Myositis ossificans and show that our case has a unique pattern.

**Conclusion:**

We report on a rare case of Myositis ossificans of the deltoid muscle and review of the literature for similar cases and MRI features of Myositis ossificans. We show that our case was unique both in presentation and MRI finding.

## Highlights

• Myositis ossificans of the deltoid muscle is extremely rare.
• We present a case with unique presentation and MRI findings.
• Our case had no history of trauma or surgery in the area.
• Our case had MRI features mimicking myxoma.
• Complete excision was curative.

## 1 Introduction

There are two main clinical entities of heterotopic ossification. The first entity is relatively common and presents as peri-articular heterotopic ossification with concurrent severe joint stiffness. This is particularly common at the elbows of burn patients [1], and at the hip following total hip arthroplasty [2]. The second entity is known as myositis ossificans. It is much less common than the first entity and presents with a well-defined mass within the muscle. This is most frequently encountered in the thigh muscles of active athletes (following intramuscular hematoma formation) and usually resolves with rest and non-steroid anti-inflammatory medications [3]. Myositis ossificans of other muscles are rare and tend to persistent and hence, the mass frequently requires surgical excision [4].

Peri-articular heterotopic ossification of the shoulder may be seen following shoulders surgery [5]. This peri-articular ossification may also be associated with a sheet of ossification within the proximal deltoid muscle or fascia [6]. In contrast, myositis ossificans presenting as well-defined mass within the deltoid is extremely rare and is frequently confused with intramuscular tumors including sarcoma.

We present a case of myositis ossificans of the deltoid and review the literature on this rare entity. Our case is unique both in presentation and findings on Magnetic Resonance Imaging (MRI). The work has been reported in line with the SCARE criteria [7].

## 2 Case report

**Fig. 1:**
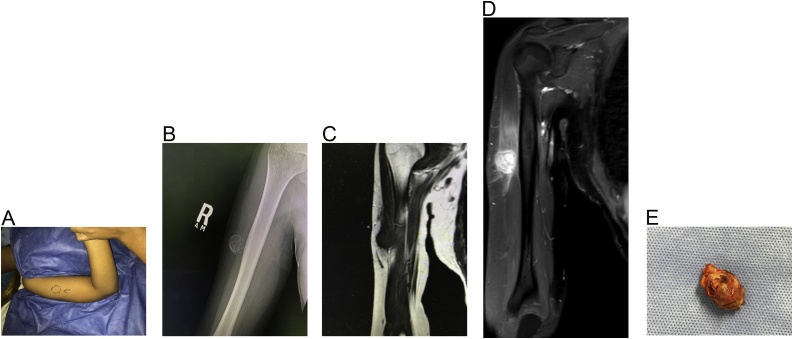
A: the site of the mass in the deltoid muscle. B: plain X-ray showing the calcified mass. C: T1 MRI image showing a hypointense lesion. D: T2 MRI image showing the mass with high intensity and internal heterogenous enhancement. E: The excised calcified mass.

**Fig. 2:**
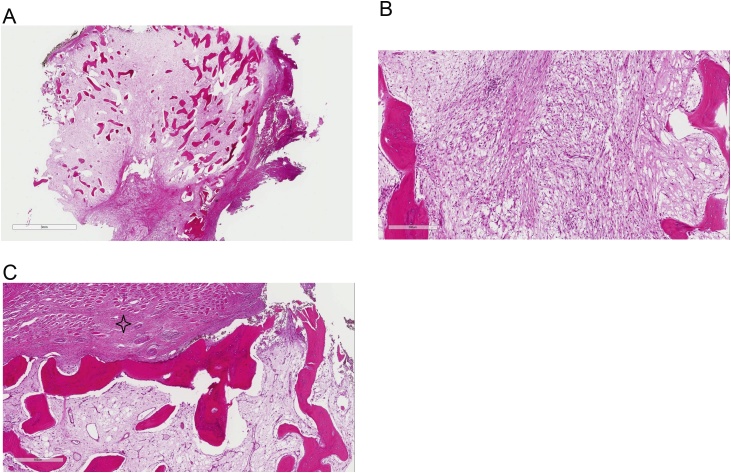
A. Low power view of the hematoxylin and eosin-stained section showing ossification within muscle with the characteristic zonation phenomenon. B. High power view showing the central portion of myositis ossificans is comprised of bland fibroblastic proliferation. C. High power view showing that towards the periphery, there is formation of mature lamellar bone. Notice the skeletal muscle fibers exterior to the lesion (asterisk).

A 21-year-old female presented with a 3-month history of a swelling in lateral aspect of the right arm. There was no history of trauma, sickness, surgery, or injection to the area. The patient was not married and had no history of pregnancy or vaccinations in the right arm. The swelling has been slowly increasing in size and was only associated with mild pain when she sleeps on her right side. Examination showed a well-defined 3 × 3 cm firm slightly tender mass. The mass was attached to the deltoid muscle. The skin was freely mobile over the mass. The overlying skin showed no erythema (Fig. 1A). There was no lymphadenopathy and the range of motion of the shoulder was normal. Routine blood tests showed no abnormalities. A plain x-ray showed a well-defined mass adjacent to the humerus with a calcified margin and spotting calcification within the mass (Fig. 1B). MRI showed a well-defined mass within the lower part of the deltoid muscle. It appeared of low-signal intensity on T1 W images (Fig. 1C) and of heterogeneous high-signal intensity on T2 images (Fig. 1D). Although myositis ossificans was considered in the differential diagnosis, the radiology report suggested that the MRI findings could represent myxoma or myxoid sarcoma. Complete surgical excision was done under general anesthesia (Fig. 1E). The mass was found to be well encapsulated within the lower part of the lateral deltoid muscle near the muscle insertion. There was no attachment to the humerus. Histopathology (Fig. 2) showed the classic picture of myositis ossificans with zonation phenomena: A central blend of fibroblastic proliferation; and towards the periphery, there was formation of osteoid and mature lamellar bone. The post-operative course was uneventful. The patient was last seen 7 months after surgery and there was no evidence of recurrence.

## 3 Discussion

Our case of myositis ossificans of the deltoid muscle is rare. Furthermore, it is unique both in presentation and in MRI findings.

We reviewed all previously reported cases of myositis ossificans presenting as a mass within the deltoid muscle [[8], [9], [10], [11]]; and these cases are summarized in Table 1. There was a total of 6 cases. One child developed the mass following an acute febrile illness; and history of arm injections (for his illness) was not specified. The remaining five cases were seen in adults with a history of trauma to the area. The site of trauma coincided with the site of the mass within the deltoid. For example, all three cases reported by Kir and Ozdemir [10] developed myositis ossificans of the deltoid just lateral to the delto-pectoral groove. All three patients were soldiers with a history of heavy military training using riffles put against the delto-pectoral groove. Similarly, the case reported by Schultzel et al. [11] had bilateral deltoid masses at the site of frequent bilateral injections of the anabolic steroids. Our case was unique, being the only case with a negative history of trauma, exercise, surgery or sickness. Hence, our case falls into the very rare category of “idiopathic” myositis ossificans [4].

**Table 1:** Cases of myositis ossificans presenting as a mass within the deltoid muscle.

Author, year of publication [reference number]	Age and sex	History of trauma exercise, or sickness	Tenderness of the mass/Shoulder movements	MRI findings	Management/outcome
Wilkes, 1976 [8]	3 years, male	Acute febrile illness	Non-tender, normal shoulder movements	Not done	Excision. No recurrence
Schmidt et al, 2001 [9]	20 years male	Nailing of the humerus	Non tender, deltoid mass with peri-articular shoulder heterotopic ossification and significant limitation of shoulder movements.	Not done	Excision with post-operative radiotherapy to the shoulder. No recurrence with regain of normal shoulder movements.
Kir and Ozdemir, 2011 [10]					
Patient #1	20 years, male	Military Training	Slightly tender, mild limitation of shoulder movements	Not done	Refused surgery with loss of follow-up
Patient #2	20 years, male	Military Training	Slightly tender, mild limitation of shoulder movements	Not done	Excision. No recurrence with regain of shoulder movement
Patient #3	20 years, male	Military Training	Slightly tender, mild limitation of shoulder movements	Not done	Excision. No recurrence with regain of shoulder movement
Schultzel et al, 2014 [11]	40 years, male	Weight lifting with frequent injections of anabolic steroids into both deltoids	Bilateral deltoid non- tender masses, normal shoulder movement	Isointense to fat signal	Excision. No recurrence
Current Case	21 years, female	No	Slightly tender, normal shoulder movement	Unique pattern (Table 2)	Excision. No recurrence

The rarity of idiopathic myositis ossificans of muscle may lead to confusion regarding the differential diagnosis. Schultzel et al. [11] reviewed the literature and stated that in the acute stage of myositis ossificans (during the first month of the disease, when ossifications are immature), periosteal osteosarcoma and synovial sarcoma must be ruled out. In the chronic stage (after 2 months, when ossifications are well established), the mature lesion of myositis ossificans may show radiological characteristics similar to other sarcomas and ossifying skeletal muscle metastases [12].

The MRI imaging of the various stages of maturation of myositis ossificans is well described in the literature [13]; and these radiological features are summarized in Table 2. Our case presented in the chronic stage. MRI findings in our case was unique (Table 2). The radiological pattern in our case mimicked the features of intramuscular myxoma/myxo-sarcoma [14]. The definitive diagnosis in these cases is based on the histological features of myositis ossificans showing the characteristic zonation phenomenon (see Fig. 2).

**Table 2:** MRI imaging features of Myositis ossificans.

Stage of disease	Intensity of the mass on MRI	
	T1 images	T2 images
Acute Stage (First month)	Intermediate signal	High signal
Subacute stage (1–2 months)	Low signal intensity at the margin of the mass with a high signal intensity in the center of the mass	Very high signal
Chronic stage (over 2 months)		
Pattern I	Isointense to fat signal	Isointense to fat signal
Pattern II	Intermediate signal	Slightly increased signal
Pattern III (seen in our case)	Low signal	High signal with internal heterogeneous enhancement

## 4 Conclusion

We report on a rare case of myositis ossificans of the deltoid muscle and review the literature for similar cases and MRI features of myositis ossificans. We show that our case was unique both in presentation and MRI findings.

## Funding

None.

## Ethical approval

The study was approved by the research committee, National Hospital (Care), Riyadh, Saudi Arabia.

## Consent

Written informed consent was obtained from the patient for publication of this case report and accompanying images. A copy of the written consent is available for review by Editor-in-chief of this Journal on request.

## Author contribution

All authors contributed significantly and in agreement with the content of the manuscript. All authors participated in data collection and in writing of the manuscript.

## Registration of research studies

Not relevant here.

## Guarantor

M M Al-Qattan.

## Provenance and peer review

Not commissioned, externally peer-reviewed.

## Declaration of Competing Interest

None.
